# Ethyl 2-Succinate-Anthraquinone Attenuates Inflammatory Response and Oxidative Stress via Regulating NLRP3 Signaling Pathway

**DOI:** 10.3389/fphar.2021.719822

**Published:** 2021-11-08

**Authors:** Burong Feng, Xiuye Zhao, Wei Zhao, Huiwei Jiang, Zijing Ren, Yingfu Chen, Ye Yuan, Zhimin Du

**Affiliations:** ^1^ Institute of Clinical Pharmacy, The Second Affiliated Hospital of Harbin Medical University (The University Key Laboratory of Drug Research, Heilongjiang Province), Harbin, China; ^2^ Department of Clinical Pharmacology, College of Pharmacy, Harbin Medical University, Harbin, China; ^3^ State Key Laboratory of Quality Research in Chinese Medicines, Macau University of Science and Technology, Macau, China

**Keywords:** Luhui derivative (LHD), inflammation, oxidative stress, NLRP3, THP-1

## Abstract

Aloe-emodin widely possesses antibacterial, anti-inflammatory, antioxidant, antiviral, and anti-infectious properties. This study investigated the effect of ethyl 2-succinate-anthraquinone (Luhui derivative, LHD) on inflammation. *In vitro*, a THP-1 macrophage inflammation model, made by 100 ng/ml phorbol-12-myristate-13-acetate (PMA) and 1 μg/ml LPS for 24 h, was constructed. The LHD group (6.25 μmol/L, 12.5 μmol/L, 25 μmol/L, 50 μmol/L) had no effect on THP-1 cell activity, and the expression of IL-6 mRNA was down-regulated in a concentration-dependent manner, of which the 25 μmol/L group had the best inhibitory effect. The migration of THP-1 macrophages induced by LPS was decreased by the LHD. Moreover, the LHD suppressed ROS fluorescence expression by inhibiting MDA expression and increasing SOD activity. *In vivo*, we revealed that the LHD, in different doses (6.25 mg/kg, 12.5 mg/kg, 25 mg/kg, 50 mg/kg), has a protective effect on stress physiological responses by assessing the body temperature of mice. Interestingly, acute lung injury (e.g., the structure of the alveoli disappeared and capillaries in the alveolar wall were dilated and congested) and liver damage (e.g., hepatocyte swelling, neutrophil infiltration, and hepatocyte apoptosis) were obviously improved at the same condition. Furthermore, we initially confirmed that the LHD can down-regulate the expression of NLRP3, IL-1β, and caspase-1 proteins, thereby mediating the NLRP3 inflammasome signaling pathway to produce anti-inflammatory effects. In conclusion, our results indicate that the LHD exerts anti-inflammatory activity via regulating the NLRP3 signaling pathway, inhibition of oxidative stress, and THP-1 macrophage migration.

## Introduction

Inflammation, a body’s response to exogenous or endogenous damage factors, is a very common and important pathological process. It has been found to mediate a wide variety of human diseases, including diabetes, cardiovascular diseases, cancer, septic shock, and sepsis. Its basic pathological changes include metamorphosis, exudation, and hyperplasia. According to the different duration, inflammation can be classified into two types: acute inflammation and chronic inflammation (Hu et al., 2014; Furtado et al., 2016; Karim et al., 2019). In addition, the inflammatory response can remove harmful substances from the body and produce a positive protective effect through initiating tissue repair. However, it should be emphasized that the inflammatory response can release a large number of inflammatory cytokines and inflammatory mediums such as IL-6, IL-1β, and iNOS; if they cannot disappear in time, it would be more severe. On the one hand, these inflammatory cytokines can disturb the balance of anti-inflammatory and pro-inflammatory reactions (Murray and Smale, 2012; Headland and Norling, 2015; Yang et al., 2016). On the other hand, the release of inflammatory factors can cause autoimmune and metabolic diseases, chronic nervous system diseases (Foster and Medzhitov, 2009; Headland and Norling, 2015), malignant tumors, and other diseases, which seriously threaten human health (Perez and Vago, 2014).

Lipopolysaccharide (LPS) is the component of the cell wall of Gram-negative bacteria (Widdrington et al., 2018), which could directly act on the cell membrane. Functionally, it can stimulate macrophages to polarize into M1-type macrophages and exude a large number of pro-inflammatory factors (Goodman and Brett, 2017; Skirecki and Cavaillon, 2019). However, by further amplifying the inflammatory response, these inflammatory factors could increase vascular permeability and inflammatory cell infiltration, thereby promoting systemic inflammation (Tandon et al., 2018). At the function level, LPS can cause systemic inflammatory response syndrome, including sepsis, shock, disseminated intravascular coagulation (DIC), and multiple organ failure (MOF) (Wang et al., 2015). Therefore, LPS is one of the important conditions to trigger an inflammatory response.

Macrophages, including classically activated macrophages (M1) and alternatively activated macrophages (M2) (Fuchs et al., 2016; Gao et al., 2017), are a kind of immune cells, which have functions of phagocytosis, antigen presentation, and releasing various cytokines, and play an important role in the physiological processes of inflammation, defense, repair, and metabolism (Su et al., 2015; Cullis et al., 2017). Furthermore, macrophage migration contributes directly to pathogen removal and wound healing (Yang et al., 2018; Carestia et al., 2019). Therefore, macrophages play a key role in mediating the inflammatory response.

**FIGURE 1:**
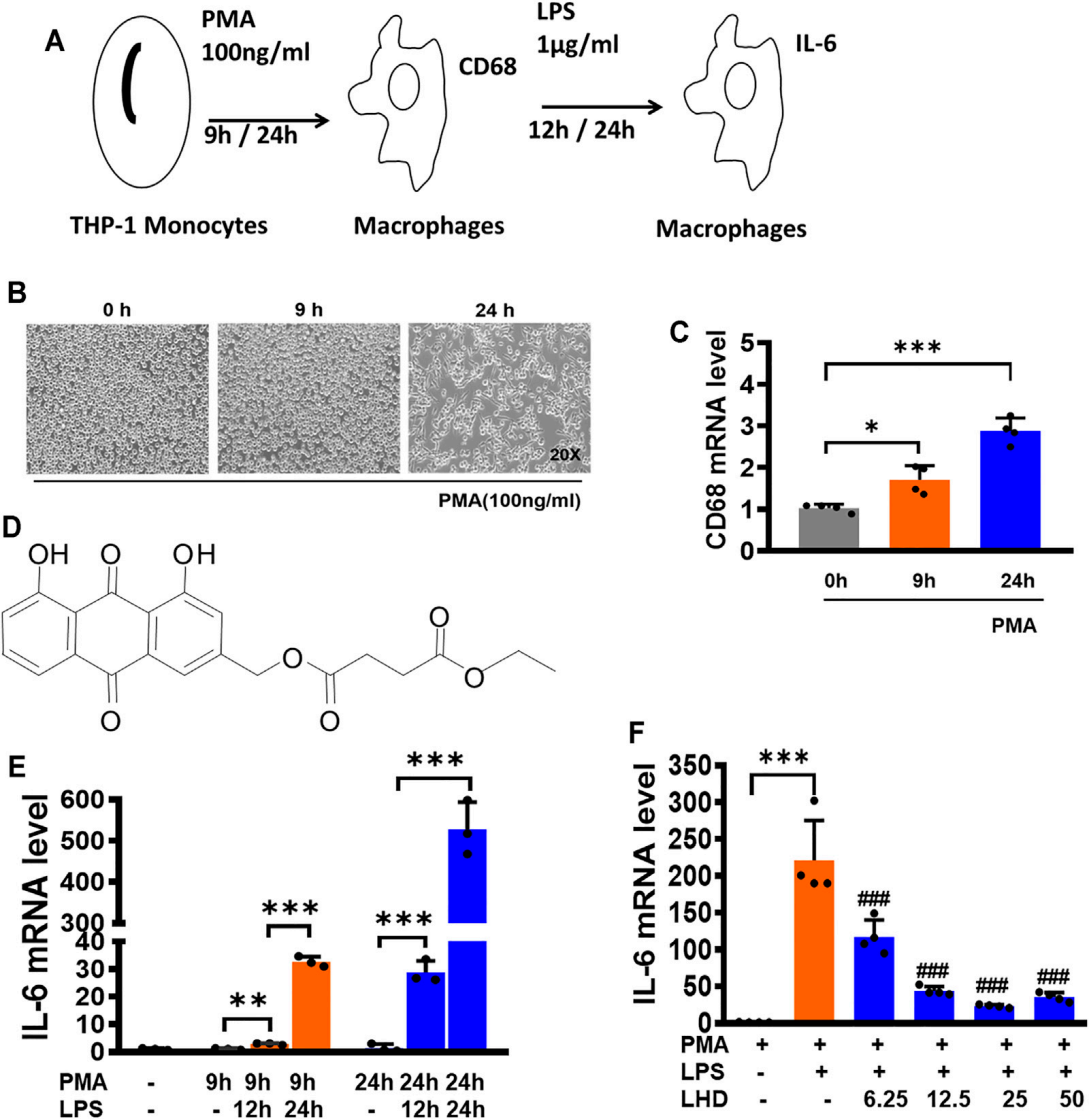
LHD can inhibit THP-1 macrophage inflammation induced by LPS. **(A)** Construction of the macrophage inflammation model. **(B)** After 100 ng/ml PMA stimulation of THP-1 cells for 0, 9, and 24 h, the morphology of THP-1 macrophages was observed under an optical microscope (20×). **(C)** After 100 ng/ml PMA stimulation of THP-1 cells for 0, 9, and 24 h, the expression of CD68 was detected by qRT-PCR, *N* = 4. **(D)** Structures of compound LHD (1,8-dihydroxy-3-(hydroxymethyl)-anthraquinone ethyl succinate). **(E)** After 1 μg/ml LPS stimulated THP-1 macrophages for 12 and 24 h, besides 100 ng/ml PMA stimulation of THP-1 cells for 0, 9, and 24 h, the expression of IL-6 was detected by qRT-PCR, *N* = 3. **(F)** After 6.25, 12.5, 25, and 50 μmol/L LHD were co-incubated with LPS- and PMA-induced THP-1 macrophages for 24 h, and the expression of IL-6 was detected by qRT-PCR, *N* = 4 (***p < 0.001, **p < 0.01 vs. the PMA group; ^#^ *p* < 0.05, ^##^ *p* < 0.01, ^###^ *p* < 0.001 vs. the LPS group).

Aloe-emodin (1,8-dihydroxyl-3-hydroxymethyl-9,10-anthraquinone) is widely found in aloe vera, rhubarb, knotweed, cassia seed, and other Chinese herbs. It is a representative anthraquinone compound of emodin type. A variety of literature studies have mentioned that emodin anthraquinone is a compound with a special structure of hydroxyl group distributed on both sides of benzene rings. Furthermore, emodin has been demonstrated to possess a series of therapeutic effects, such as antibacterial, anti-inflammatory, antioxidant, antiviral, and anti-infectious effects (Mu and Zhi-min, 2015). Arosio et al. confirmed that aloe-emodin could inhibit the inflammatory response after lipid peroxidation by reducing the expression level of TNF-α mRNA (Alshatwi and Subash-Babu, 2016). However, emodin has been reported to exert toxicity and decrease absorption, so it has not been directly used in clinical trials. Therefore, it is necessary to optimize the structure of emodin anthraquinone compounds. In this study, we reported the finding that the Luhui derivative exhibits (Figure 1D) its anti-inflammatory effects by mediating the NLRP3 inflammasome signaling pathway and inhibits inflammatory cytokine expression in serum.

## Materials and Methods

### Reagents

LPS (a surface glycolipid produced by Gram-negative bacteria) was purchased from Sigma-Aldrich (St. Louis, MO), and stock solutions of 1 mg/mL were prepared and stored at −20°C. Ethyl 2-succinate-anthraquinone (Luhui derivative, LHD) is a novel compound monomer formed by covalent binding of monomethyl succinate to the anthraquinone mother nucleus of aloe-emodin using chemical synthesis techniques in our laboratory. Kits of MDA and SOD were purchased from the Nanjing Jian Cheng Bioengineering Institute. IL-6, IL-1β, and IL-18 ELISA kits were purchased from the Neobioscience Institute (Shenzhen, China) and Elabscience Institute (Wuhan, China). The antibodies used in this study with their working dilutions are indicated in parentheses: NLRP3 (1:500) was purchased from the Wanle Institute, IL-1β (1:500) was purchased from the Elabscience Institute, and caspase-1 (1:500) and ASC (1:500) were purchased from Proteintech Group, Inc. (Wuhan, China).

### Animal Model

C57BL/6 mice (18–22 g) were provided by the Animal Center of the Second Affiliated Hospital of Harbin Medical University. All the animals were involved in the experiment with the approval of the Ethics Committee of Harbin Medical University (SYDW2019-258), and the experimental procedures were performed in accordance with the National Institutes of Health (NIH) Guide for the Care and Use of Laboratory Animals.

After 3 days of adaptive feeding, according to the random number table method, 56 male C57BL/6 mice were randomly divided into seven groups (control group, model group, LHD 6.25 mg/kg, LHD 12.5 mg/kg, LHD 25 mg/kg, LHD 50 mg/kg, and dextromethorphan (DXM) 5 mg/kg group), and there were eight mice in each group. Then, the mice in each group were given the drug by gavage at the experimental concentration for six consecutive days (the blank control group and the model group were given the same amount of physiological salt), and the weight changes of the mice were recorded within 6 days. One hour after gavage, each group was injected with normal saline and LPS, respectively, to construct the model of inflammation *in vivo*. The body temperature of the mice in each group was measured with a frontal tester 6 h after LPS injection. Subsequently, orbital blood was taken from mice to detect the changes of IL-6, IL-1β, and IL-18 inflammatory factors by ELISA.

### Cell Culture

The human myeloid leukemia mononuclear cell line (THP-1) was obtained from the GEFAN Biotechnology company (Shanghai, China) and cultured in RPMI 1640 medium (Corning, United States) supplemented with 10% fetal bovine serum (ExCell Bio, China). The THP-1 cells were cultured at 37°C with 5% CO_2_ and 95% air after their isolation.

### Histological Analyses

6 h after LPS injection, the whole lung tissue was fixed with 10% formaldehyde for 24 h, followed by gradient dehydration with sucrose and embedding in paraffin. Next, the tissue block was cut into four-bedroom slices and placed at 65°C for overnight baking. Then, xylene, anhydrous ethanol, 90% ethanol, 70% ethanol, and distilled water were used for dewaxing. Hematoxylin staining solution was used for staining, and neutral gum was used to seal the tablet. Pathological changes of lung and liver tissues were observed and photographed under a light microscope.

### Cell Counting Kit-8

THP-1 cells were adhered by 100 ng/ml PMA in a 96-well plate (1 × 10^4^ per well) for 24 h. Then, different doses of LHD (6.25, 12.5, 25, and 50 µmol/L) were added to the 96-well plate. Finally, CCK-8 (10 μl) was added to each well and kept in an incubator for 4 h (MedChemExpress, China). The culture plates were shaken for 30 s, and the absorbance was recorded at 450 nm. The wavelength was read by an Infinite M200 microplate reader (Tecan, Salzburg, Austria).

### Quantitative Real-Time Polymerase Chain Reaction

Total RNA samples were extracted from cells using Trizol reagent (Invitrogen, United States), and quantitative PCR and reverse transcription were performed following the manufacturer’s protocols. RNA quantity was detected with the NanoDrop TM 8000 spectrophotometer (Thermo Scientific, France). Sequences of gene-specific PCR primers (Shanghai Generay Biotech Co., Ltd., China) used were as follows:

**Table:** 

Human CD68	Forward: 5′-CTACATGGCGGTGGAGTACAA-3′
	Reverse: 5′-ATGATGAGAGGCAGCAAGATGG-3′
Human IL-6	Forward: 5′-GGTACATCCTCGACGGCATCT-3′
	Reverse: 5′-GTGCCTCTTTGCTTTCAC-3′
Human β-actin	Forward: 5′-AGCCTCGCCTTTGCCGA-3′
	Reverse: 5′-CTGGTGCCTGGGGCG-3′

Quantitative PCR was performed in 20 μl volumes with SYBR Green PCR Master Mix (Roche, United States) at 95°C for 10 min and 40 cycles at 95°C for 15 s, 60°C for 30 s, and 72°C for 30 s with Light Cycler 480 (Roche, United States). IL-1β, IL-6, and TNF-α levels were calculated according to the 2^−ΔΔCt^ method, and GAPDH mRNA was measured as an internal control.

### Western Blot

Total proteins were extracted from THP-1 cells. The cells were lysed with 40 μl of RIPA buffer (Beyotime, Jiangsu, China), and then the protein content was measured with the Bicinchoninic Acid (BCA) Protein Assay Kit (Bio-Rad, Mississauga, ON, Canada). Protein samples (60–90 μg) were separated by sodium dodecyl sulfate–polyacrylamide gel electrophoresis (SDS-PAGE) (10–15%) at 70 V for 30 min and 100 V for 1.5 h and then transferred into nitrocellulose membranes and blocked with 5% non-fat milk for 2 h at room temperature. The membranes were incubated with primary antibodies (NLRP3, ASC, IL-1, caspase-1, and GAPDH) in a shaker at 4°C overnight. The strips were washed with PBST (PBS containing 0.1% Tween-20) and incubated with IRDye secondary antibody (LI-COR, United States) for 1 h. The protein expression was measured by the Odyssey CLx Infrared Imaging System (LI-COR Biosciences, Lincoln, NE, United States). The bands were quantified by measuring the band intensity using Odyssey CLx version 2.1.

### Cell Scratch Experiment

First, each hole in the six-well plate was covered with cells (5 × 10^5^ cells). Then, the spear head was placed perpendicular to the cell plane, and scratches were made on the cell layer along the top of the six-well plate. The scratches were kept vertical when the pipette head slid down to the bottom, and it is ensured that the width of each scratch is the same. After the scratch was completed, the cells were washed with sterile PBS three times to remove the non-adherent cells, which could ensure the gap left after the scratch could be clearly seen. Then, the fresh RPMI 1640 medium was replaced, the cells were cultured in a 37°C 5% CO_2_ incubator, and the cells were taken out at 0, 24, and 48 h, respectively. The changes of scratch width in each group were observed and recorded under an inverted microscope.

### ROS Assessment

Aerobic cells produce a series of reactive oxygen species in the process of metabolism. DCFH-DA is a non-fluorescent probe that can penetrate into cells. The fluorescence intensity of DCFH-DA represents the changes in the level of reactive oxygen species in cells. THP-1 cells were incubated with 10 μM DCFH-DA in a cell culture box of 5% CO_2_ at 37°C for 20 min. Then, the cells were washed with serum-free RPMI 1640 medium three times to fully wash DCFH-DA from the cell surface in the six-well plate. Next, the cells were fixed with 4% paraformaldehyde and washed with PBS three times. Finally, DAPI was used to stain nuclei for 15 min. An FV1000 laser confocal microscope was used to detect the fluorescence intensity of reactive oxygen species at the excitation wavelength of 488 nm and the emission wavelength of 525 nm.

### Malondialdehyde and Superoxide Dismutase Content Assessment

THP-1 cells were cultured in six-well plates. Then, the differentiation of THP-1 cells into macrophages was induced by addition of 100 ng/ml PMA. After 24 h, THP-1 macrophages were stimulated by 1 μg/ml LPS to construct an inflammatory model. Finally, different doses of LHD (6.25 µmol/L, 12.5 µmol/L, 25 µmol/L, 50 µmol/L) were added to co-stimulate the culture with 1 μg/ml LPS for 24 h. THP-1 macrophages were digested by 0.25% trypsin solution. After centrifugation, the cells were broken by sonication on ice, and the protein concentration was measured and calculated by BCA. MDA and SOD contents were determined by a commercial ELISA kit according to the manufacturer’s instructions.

### Inflammatory Cytokines Analyses

We used ELISA to economically detect the expression of IL-6 and other inflammation. The animals were anesthetized, and blood samples were collected via orbital puncture. Subsequently, the blood samples were maintained at room temperature for 20 min and centrifuged (3,000 rpm/min) at 4°C for 5 min. All the samples were preserved at −80°C. The ELISA kits were used to measure the level of IL-6 and other inflammation in mice serum after administering different doses of LHD (6.25 µmol/L, 12.5 µmol/L, 25 µmol/L, 50 µmol/L) according to the manufacturer’s instructions.

### Data Statistical Analyses

All the measurement data involved in this experiment were expressed as mean ± SEM. One-way ANOVA was used to distinguish the data of three or more groups. *p < 0.05, **p < 0.01, and ***p < 0.001 showed statistical differences. *p* > 0.05 indicated no statistical difference. The data analysis and drawing involved in this experiment were processed by GraphPad Prism version 8.0 (GraphPad Software, United States).

## Results

### Construction of THP-1 Macrophage Inflammation Model

THP-1 cells were induced with 100 ng/mL phorbol-12- myristate-13-acetate (PMA) for 0, 9, and 24 h (Reeves et al., 2015). At 0 h, the cells were suspended. At 9 h, some cells showed adherent state but no pseudopodia. At 24 h, the cells were all adherent to the wall, and most of them had pseudopodia sticking out, suggesting that THP-1 cells were differentiated into macrophages (Figure 1A) and the cell morphology at different time points was observed under a microscope (Figure 1B). Then, the expression of CD68 mRNA was detected by qRT-PCR. Compared to that at 0 h, the increase of CD68 mRNA was the most obvious at 24 h, which was consistent with the observation results of cell morphology (Figure 1C). In order to detect the effect of LPS on the release of inflammatory cytokines (Ávila-Román et al., 2018), THP-1 macrophages were induced with 1 μg/ml LPS for 12 h and 24 h and used qRT-PCR to detect the expression of IL-6 mRNA (Figure 1E). After LPS treatment for 12 and 24 h, the expression of IL-6 mRNA was significantly increased compared to that in the PMA group, and after LPS treatment for 24 h, the expression of IL-6 mRNA was nearly 500 times higher than that of the PMA group. Therefore, we selected 1 μg/ml LPS to stimulate the THP-1 macrophages for 24 h to conduct the following experiments.

### Inhibitory Effect of Luhui Derivative on THP-1 Macrophage Release of Inflammatory Factors

We next explored whether the LHD impacted the THP-1 macrophage release of inflammatory factors. Before we assayed inflammatory factors, a CCK-8 assay was performed to reveal that the LHD had no effect on cell activity after co-incubation for 24 h at 6.25, 12.5, 25, and 50 μmol/L (Supplementary Figure S1). Next, the effects of LHD on the release of inflammatory cytokines by THP-1 macrophages were detected by qRT-PCR. Compared to that in the LPS group, the expression of IL-6 mRNA in the groups with different doses of LHD was significantly reduced, and the three doses of 6.25, 12.5, and 25 μmol/L showed concentration dependence, among which 25 μmol/L showed the most obvious inhibitory effect (Figure 1F). In conclusion, 25 μmol/L LHD could inhibit the THP-1 macrophage release of inflammatory factors.

### Inhibitory Effect of Luhui Derivative on THP-1 Macrophage Migration Function

**FIGURE 2:**
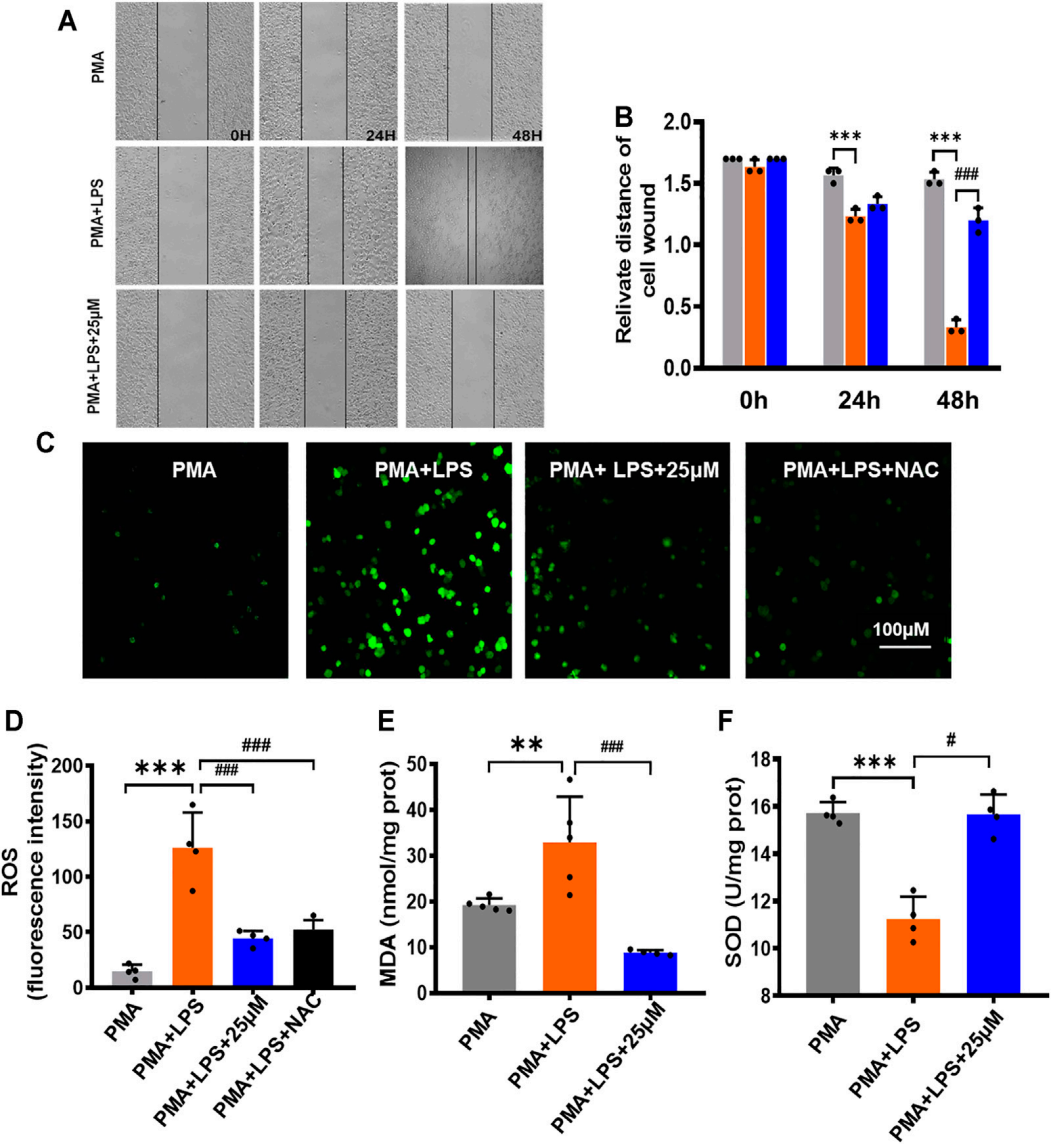
Inhibitory effect of LHD on the oxidative stress and inflammatory cytokines in THP-1 macrophages. **(A)** 25 µM LHD was co-incubated with LPS-induced THP-1 macrophages for 0, 24, and 48 h, and the cell migration capacity was detected by a cell scratch assay (20×). **(B)** Cell scratch width (cm), *N* = 3. **(C)** Representative graph of ROS levels (200×). Scale = 100 mΜ. **(D)** Statistical graph of ROS levels, *N* = 5. **(E)** SOD activity, *N* = 5. **(F)** MDA content, *N* = 4 (***p < 0.001, **p < 0.01 vs. the PMA group; ^#^ *p* < 0.05, ^##^ *p* < 0.01, ^###^ *p* < 0.001 vs. the LPS group).

The migration of macrophages plays a positive role in pathogen clearance and wound healing. The relative migration distance of cells at each time point was observed by a scratch experiment, and the results are shown in Figures 2A,B. The inhibitory effect of the LPS+25 μmol/L LHD group on lateral cell migration was most obvious at 48 h and showed that the LHD could inhibit the expression of the inflammatory response by inhibiting the migration of macrophages.

### Inhibitory Effect of Luhui Derivative on the Oxidative Stress and Inflammatory Cytokines in THP-1 Macrophages

Inflammatory reactions can lead to an excessive increase in free radicals and superoxide anions. When THP-1 macrophages were treated with LPS for 24 h and treated with LPS+25 μmol/L LHD for 24 h at the same time, compared to that in the PMA group, the total fluorescence expression of ROS in the LPS group increased (Figures 2C,D). However, other relevant indicators of oxidative stress were expressed as follows: MDA content increased statistically, but SOD activity decreased statistically. Compared to the LPS group, the LPS+25 μmol/L LHD group had an inhibitory effect on the level of oxidative stress, which was reflected in the decrease of total ROS fluorescence expression, the decrease of MDA content, and the increase of SOD activity (Figures 2E,F).

### Effects of Luhui Derivative on Animal Models of Myocardial Infarction

**FIGURE 3:**
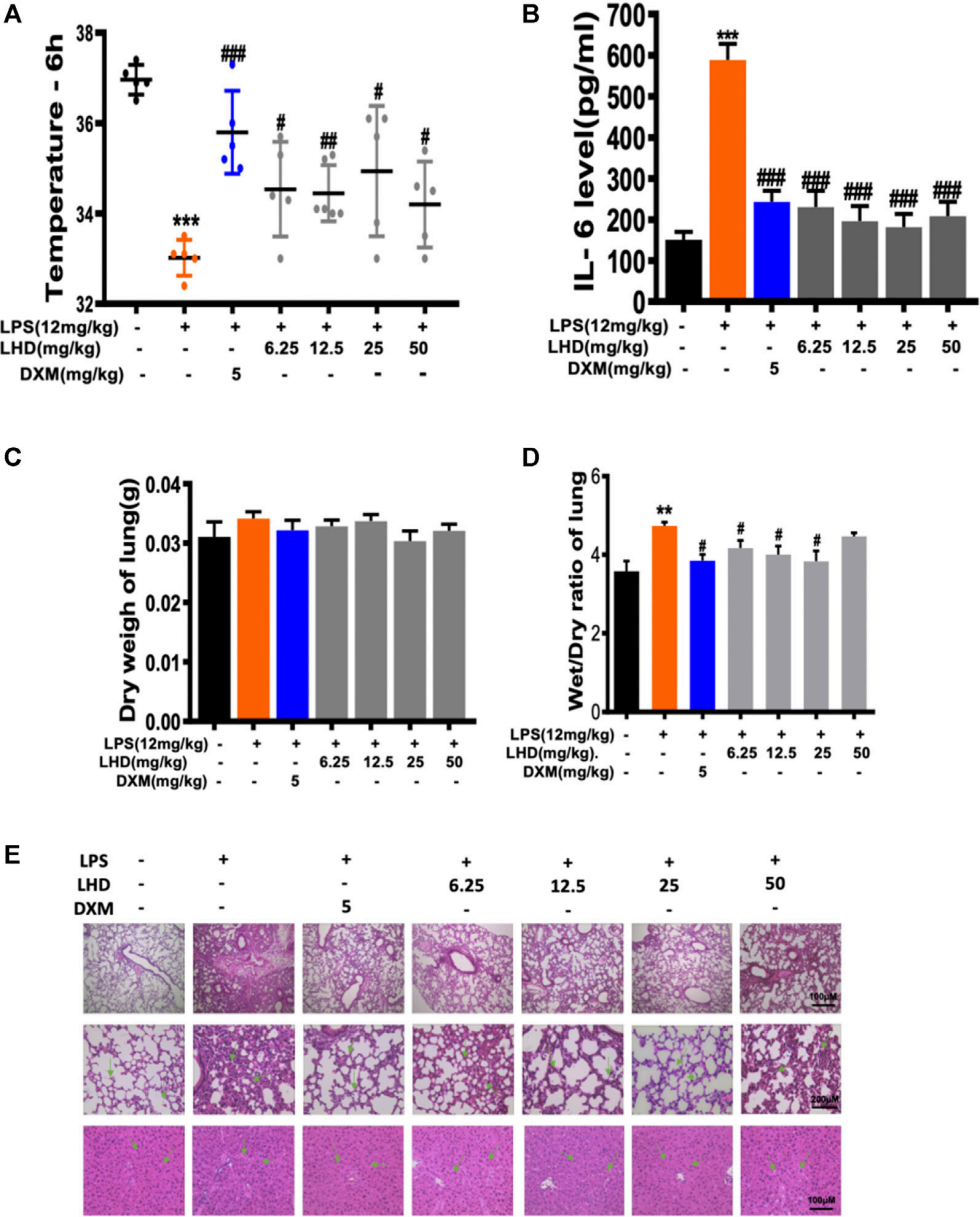
Inhibitory effect of LHD on LPS inflammation *in vivo*. **(A**) Temperature record of mice after intraperitoneal injection of 12 mg/kg LPS for 6 h, besides the intragastric administration of different concentrations of LHD or 5 mg/kg DXM for 6 days, *N* = 6. **(B)** The IL-6 inflammatory cytokine was detected by ELISA. **(C)** Lung dry weight of mice, *N* = 6. **(D)** Lung wet–dry weight ratio of mice, *N* = 6. **(E)** H&E staining results of lung tissue and liver tissue, *N* = 6 (***p < 0.001, **p < 0.01 vs. the PMA group; ^#^ *p* < 0.05, ^##^ *p* < 0.01, ^###^ *p* < 0.001 vs. the LPS group).

First, we assessed the average body temperature of each group of mice after treatment with different doses (6.25 mg/kg, 12.5 mg/kg, 25 mg/kg, 50 mg/kg) of LHD; compared to the normal body temperature at 37°C, it did not change much. The average body temperature of mice in the 25 mg/kg group and the dextromethorphan (DXM) group was maintained at about 35°C, and the temperature of mice in the other concentration of the LHD group was maintained at about 34.5°C. The results showed that the LHD has a protective effect against stress physiological responses (Figure 3A). Next, we detected the changes of inflammatory cytokine IL-6 in the serum of mice in each group by ELISA (Figure 3B). It has been reported that LPS can cause multiple organ failure and even the death of the patient by triggering tissue infusion and increased release of pro-inflammatory mediators. So, in contrast with the control group, the LPS group had severe pulmonary edema by measuring the lung wet–dry weight ratio and body weight of mice (Figures 3C,D and Supplementary Figure S2). In addition, by observing lung tissue sections under the microscope, we found that the normal structure of the alveoli in the LPS group had disappeared, the capillaries in the alveolar wall were dilated and congested, and the edema of the alveolar interval was widened, accompanied by inflammatory cell infiltration, indicating that the LPS group had acute lung injury. Moreover, we revealed that the LPS group had hepatocyte swelling, neutrophil infiltration, and hepatocyte apoptosis on the liver tissue. However, when given different doses (6.25 mg/kg, 12.5 mg/kg, 25 mg/kg, 50 mg/kg) of LHD, the above-mentioned injury symptoms of lung and liver tissues in each group were significantly improved (Figure 3E).

### Luhui Derivative Regulates the NLRP3 Inflammasome Signaling Pathway

**FIGURE 4:**
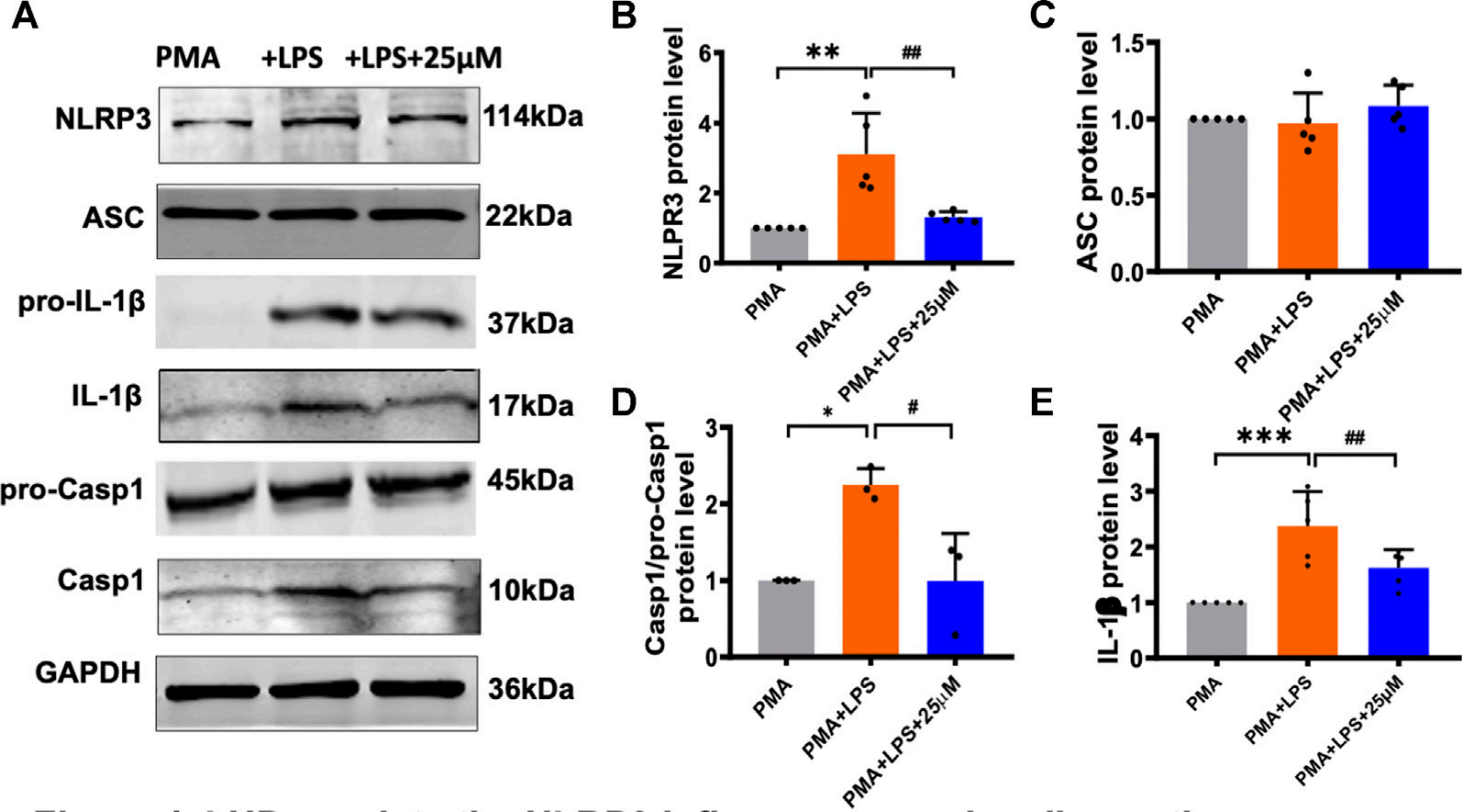
LHD regulates the NLRP3 inflammasome signaling pathway. The THP-1 cells were pre-treated with 1 μg/ml LPS for 24 h and subjected to 25 μmol/L LHD for 24 h, and then the expression of NLRP3, ASC, caspase-1, and IL-1β proteins was detected by western blot analysis. **(A)** Protein representation diagram. **(B)** Statistical map of NLRP3 protein level (*N* = 5; **p < 0.001 vs. the PMA group; ^##^ *p* < 0.01 vs. the LPS group). **(C)** Statistical graph of ASC protein. **(D)** Statistical map of caspase-1 protein level (*N* = 3; *p < 0.05 vs. the PMA group; ^#^ *p* < 0.05 vs. the LPS group). **(E)** Statistical map of IL-1β protein levels (*N* = 5; ***p < 0.001 vs. the PMA group; ^##^ *p* < 0.01 vs. the LPS group).

According to reports in the literature, NLRP3 interacts with ASC and caspase-1 to activate the NLRP3 inflammasome and then cleaves the immature inflammatory factor pro-IL-1β to mature and secrete it extracellularly, causing a series of inflammatory reactions. Next, we performed western blot to detect that the expression level of related protein in the LPS model group and LHD group (Figure 4A). The expression level of NLRP3 protein in the LPS model group was up- regulated, while the expression level of NLRP3 protein was significantly down-regulated after treatment with 25 µmol/L LHD (Figure 4B). In contrast, the expression level of ASC protein did not change significantly before and after LPS or LHD administration (Figure 4C). Additionally, compared to those in the PMA group, the expression levels of IL-1β and caspase-1 proteins in the LPS group were up-regulated, and the expression levels of IL-1β and caspase-1 proteins were significantly down-regulated after treatment with 25 µmol/L LHD. However, pro-caspase and pro-IL-1β protein expression levels did not change significantly before and after LPS or LHD administration (Figures 4D,E). The results showed that the LHD down-regulated the expression of NLRP3, IL-1β, and caspase-1 proteins, thereby mediating the NLRP3 inflammasome signaling pathway to exert anti-inflammatory effects.

### Inhibitory Effect of Luhui Derivative on Inflammatory Cytokines in THP-1 Macrophages and Serum

**FIGURE 5:**
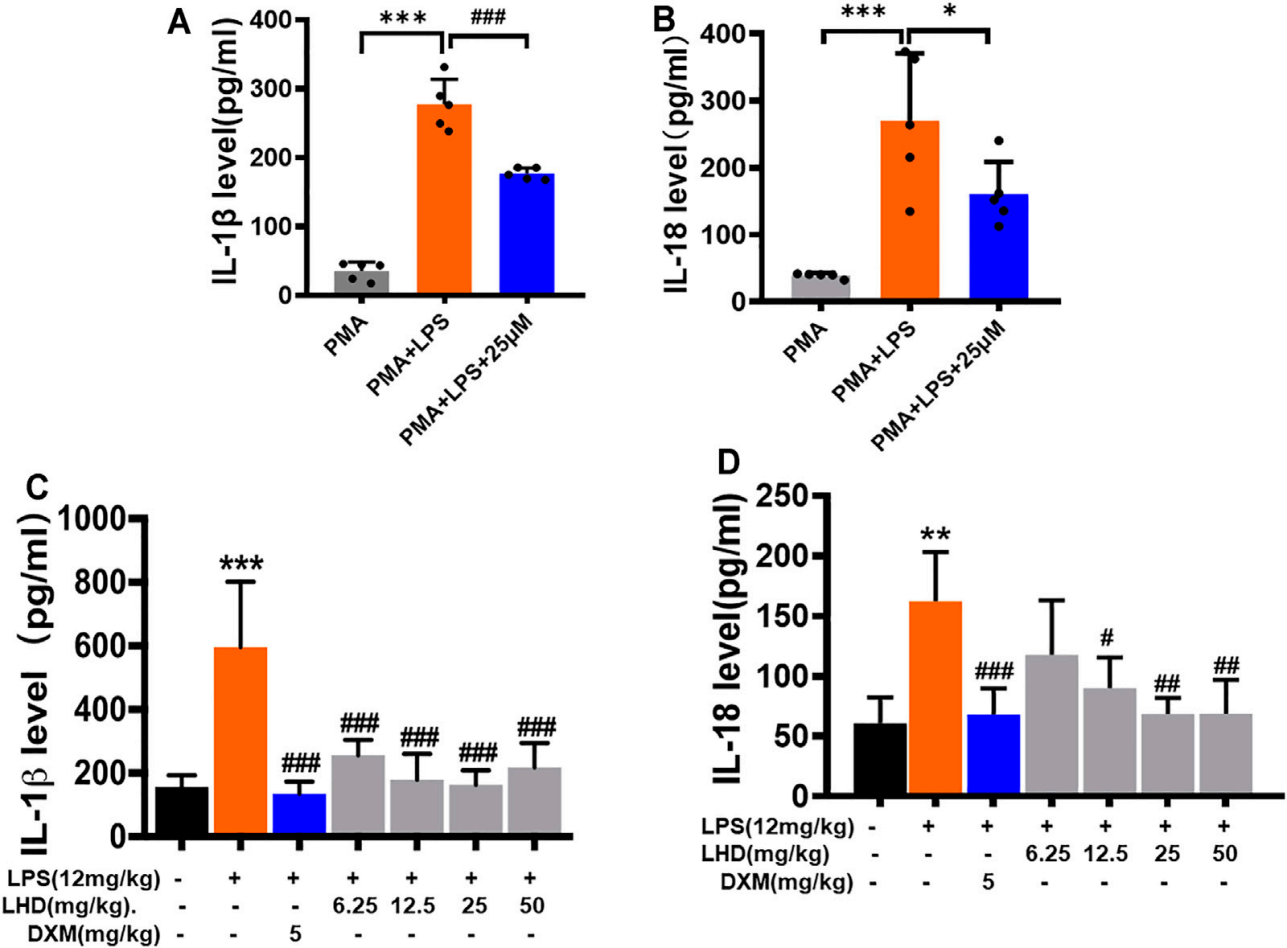
Inhibitory effect of LHD on inflammatory cytokines in serum. **(A,B)** IL-1β and IL-18 inflammatory cytokines were detected by ELISA, *N* = 5. **(C,D)** The different concentrations of LHD were co-incubated with LPS-induced THP-1 macrophages for 24 h, and the expression of IL-1β and IL-18 was detected by ELISA, *N* = 4 (***p < 0.001, **p < 0.01 vs. the PMA group; ^#^ *p* < 0.05, ^##^ *p* < 0.01, ^###^ *p* < 0.001 vs. the LPS group).

The release of a large number of inflammatory cytokines and inflammatory mediators can lead to the occurrence of inflammatory diseases, such as diabetes, autoimmune diseases, and sepsis. Therefore, we investigated the changes of inflammatory cytokines (IL-6, IL-18, and IL-1β) in THP-1 macrophages and serum of mice in each group by ELISA. The results showed that, after THP-1 macrophages were treated with LPS for 24 h, compared to that in the PMA group, the expression of inflammatory cytokines (IL-18 and IL-1β) significantly increased. However, the IL-18 and IL-1β expression levels were reduced when THP-1 macrophages were treated with LPS+25 μmol/L LHD for 24 h at the same time (Figures 5A,B). Next, we detected the content of inflammatory cytokines in the serum of mice. The results indicated that the LPS group was significantly higher than the control group after being infected with LPS for 6 h. On the contrary, compared to that in the LPS group, the expression of inflammatory cytokines in the serum of each group decreased significantly at different doses of LHD (6.25 mg/kg, 12.5 mg/kg, 25 mg/kg, 50 mg/kg) (Figures 5C,D). In conclusion, the results showed that the LHD could inhibit the high expression of inflammatory cytokines caused by LPS.

## Discussion

Inflammatory response is an important immune defense mechanism in the human body, which helps the body to resist pathogenic microbial infection (Medzhitov, 2008). However, the imbalance of inflammatory response can also lead to tissue and organ damage, thus promoting the occurrence of diseases. Inflammatory responses can be stimulated by a variety of sensors, for example, pathogenic microorganisms can stimulate the TLR (Toll-like receptor) family to mediate the inflammatory response (Ko et al., 2020). In addition, physical, chemical, and other harmful stimuli can also induce the secretion of inflammatory mediators and cytokines in the body via mediating pressure receptors, temperature receptors, and other signal transduction to cells, so as to induce the body to produce an inflammatory response. In this study, we uncovered a functional role of LHD inhibiting the THP-1 macrophage release of inflammatory factors via regulating the NLRP3 inflammasome signaling pathway.

It has been reported that aloe-emodin has antibacterial (Ji et al., 2020), antiviral (Xu et al., 2021), hepatoprotective (Zheng et al., 2019), anti-inflammatory (Chen et al., 2020), and anticancer (Galiardi-Campoy et al., 2021) pharmacological functions and has a broad clinical application prospect. For example, aloe-emodin inhibits the growth of A375.S2 cells by inhibiting the mRNA expression and enzyme activity of N-acetyltransferase (Lin et al., 2005). Tao et al. also found that aloe-emodin plays a neuroprotective role in Alzheimer’s disease by reducing the release of NO, lactate dehydrogenase, and intracellular ROS to regulate oxidative stress response (Tao et al., 2014). However, long-term and high-dose use of aloe-emodin may result in high toxicity, poor intestinal absorption, short half-life, and low bioavailability. For example, Vath et al. showed that exposure of human skin fibroblasts to aloe-emodin and ultraviolet radiation causes significant phototoxicity (Vath et al., 2002). Zaffaroni et al. also showed that, after intraperitoneal injection of aloe-emodin at a dose of 20 mg/kg, it rapidly distributes and disappears from the plasma, when a terminal half-life of 78 min was measured (Zaffaroni et al., 2003). Therefore, further optimization is needed to improve the problem of high toxicity of emodin anthraquinone-type compounds.

NLRP3 inflammasome is a novel high-molecular-weight protein complex whose activation can promote the process of inflammation (Kelley et al., 2019). In recent years, relevant studies have shown that it is involved in the occurrence of a variety of major human diseases such as type 2 diabetes (Rovira-Llopis et al., 2018), gout (Goldberg et al., 2017), Parkinson’s disease (Wang et al., 2019), and fatty liver disease (Zhang et al., 2018). Therefore, it may become a potential intervention target of the above diseases. When NLRP3 inflammasome activation is inhibited, its secretion and release of inflammatory factors such as IL-1β and IL-18 are reduced to play an anti-inflammatory role. Therefore, activation of NLRP3 inflammasome becomes a new signaling pathway to prevent and treat inflammatory diseases. It has been reported in the literature that knockout of NLRP3 gene not only can inhibit the activation of NLRP3 inflammasomes *in vivo* but also can reduce the septic shock caused by LPS (Xu et al., 2018). Based on the above study, we detected LPS-induced NLRP3 inflammasome activation at the macrophage level and determined that the LHD plays a role in regulating NLRP3 inflammasomes by down-regulating NLRP3, caspase-1, and IL-1β protein expressions. At the same time, we predicted the target of LHD on NLRP3 by AutoDock software, and subsequent experiments are required to verify the specific role of its target.

On the one hand, our results showed that 25 μM of LHD significantly inhibited cell migration and IL-1β and IL-18 expressions *in vitro*. Thus, the LHD can play an anti-inflammatory role by inhibiting macrophage migration and inflammatory cytokine expression. On the other hand, 25 μM LHD can also significantly inhibit ROS and MDA levels and increase SOD activity, showing the inhibitory effect of LHD on oxidative stress response, and can also inhibit the increase of IL-6 inflammatory factor expression caused by LPS in a dose-dependent manner. *In vivo*, different doses of LHD (6.25, 12.5, 25, and 50 mg/kg) can reduce the increase of IL-6, IL-1β and IL-18 inflammatory factors in serum and the increase of lung wet-to-dry weight ratio in LPS model after preventive treatment, and the three dose groups of 6.25, 12.5, and 25 mg/kg showed a dose dependent trend, and the decrease was more significant with the increase of dose. At the same time, different doses of LHD can also significantly alleviate the pathological injury of lung and liver tissues and the physiological reaction of hypothermia. It is worth mentioning that, in this study, the low dose of LHD was observed at 6.25 μM or 6.25 mg/kg. Again, the anti-inflammatory effects of emodin derivatives of aloe vera *in vitro* and *in vivo* are described.

The LHD is a new compound which has been chemically modified. It has better anti-inflammatory and antibacterial activity, lower toxicity, and easier absorption than aloe-emodin. However, whether the LHD can specifically inhibit the activation of NLRP3 inflammasomes remains to be further studied in subsequent experiments. Clarifying the specific relationship between the LHD and NLRP3 inflammasomes will provide new ideas for the research and development of other anti-inflammatory drugs.

## Data Availability

The raw data supporting the conclusions of this article will be made available by the authors, without undue reservation.
