# Supplementary figures and images for: Ethyl 2-Succinate-Anthraquinone Attenuates Inflammatory Response and Oxidative Stress via Regulating NLRP3 Signaling Pathway
Source: Front Pharmacol. 2021 Nov 8;12:719822. doi: 10.3389/fphar.2021.719822 (PMC8607229; doi:10.3389/fphar.2021.719822)

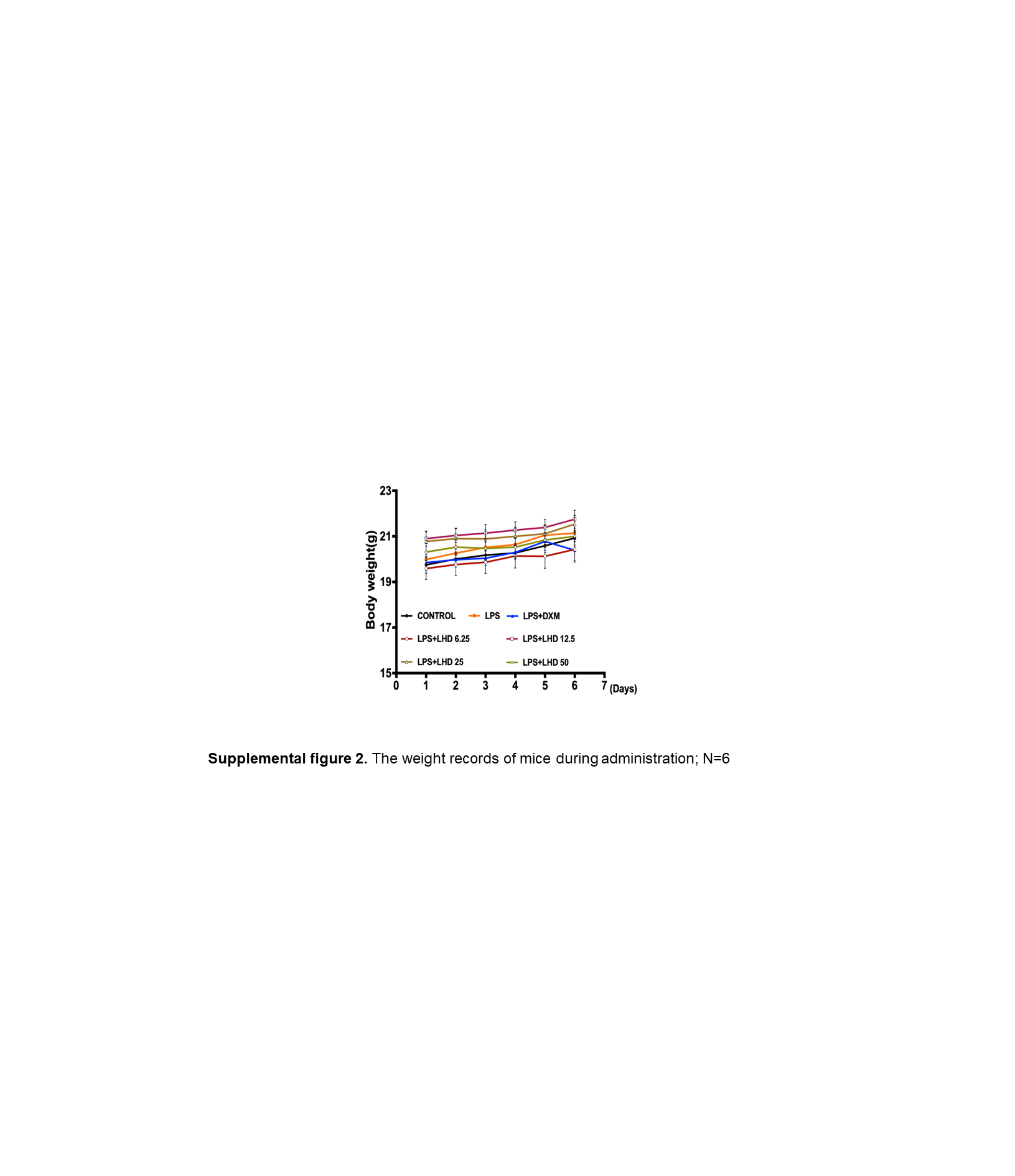

Supplement: Supplementary file 1 [file Image2.TIF]

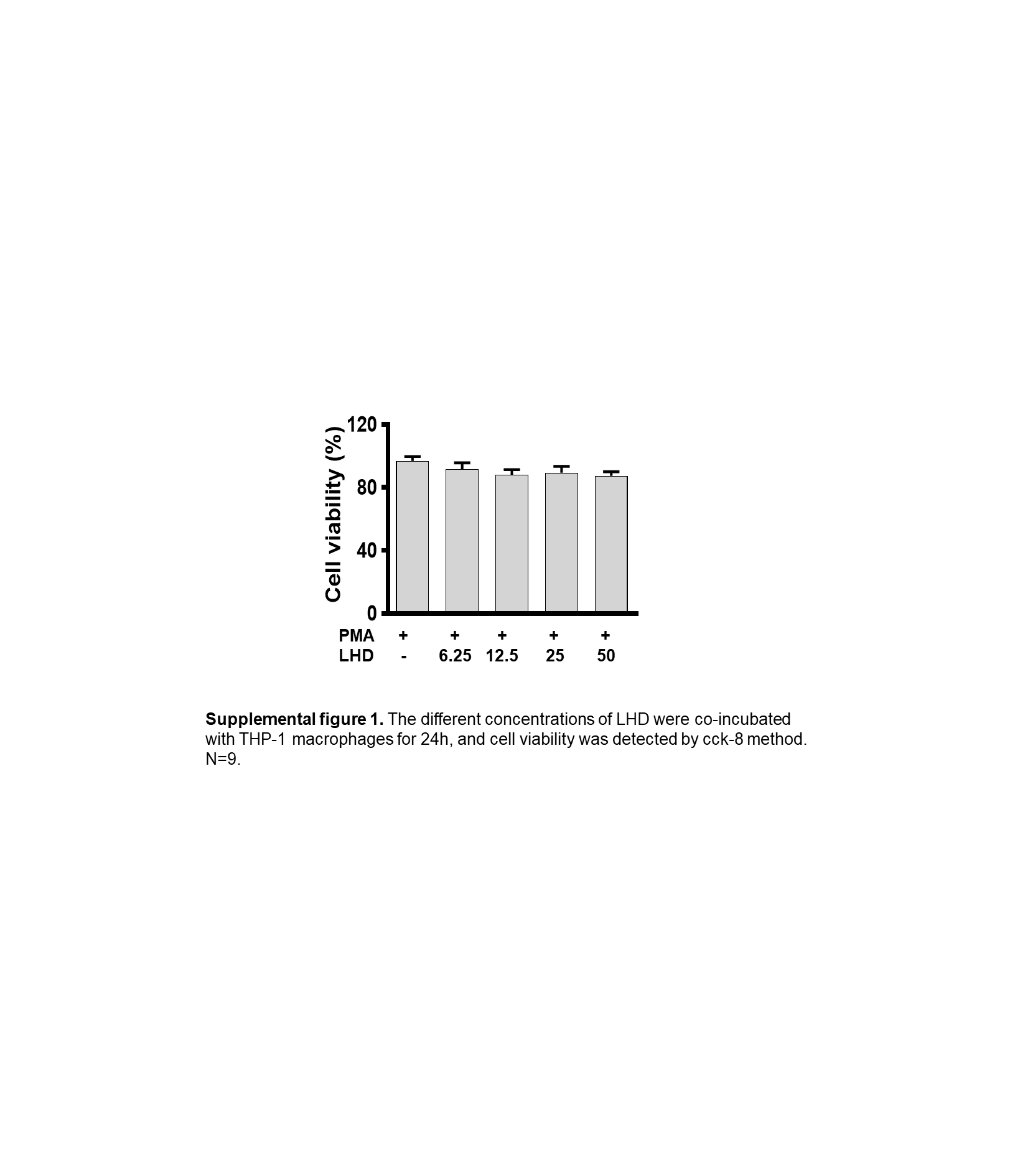

Supplement: Supplementary file 2 [file Image1.TIF]
